# The Use of HPLC-PDA in Determining Nicotine and Nicotine-Related Alkaloids from E-Liquids: A Comparison of Five E-Liquid Brands Purchased Locally

**DOI:** 10.3390/ijerph16173015

**Published:** 2019-08-21

**Authors:** Dominic Palazzolo, John M. Nelson, Zuri Hudson

**Affiliations:** 1 Department of Physiology, DeBusk College of Osteopathic Medicine, Lincoln Memorial University, Harrogate, TN 37752, USA; 2 Department of Biology, School of Mathematics and Sciences, Lincoln Memorial University, Harrogate, TN 37752, USA

**Keywords:** HPLC, ECIGs, E-liquids, aerosol, cigarettes, smoke, nicotine, nicotine-related alkaloids

## Abstract

E-liquid manufacturers are under scrutiny concerning the purity and concentration accuracy of nicotine and the minor nicotine-related alkaloids (NRAs) packaged in their products. In this communication we report concentrations of nicotine and five NRAs (nornicotine, cotinine, anabasine, anatabine, myosmine) from locally purchased E-liquids. Methods: Five brands of E-liquids (three bottles each) were purchased locally. Additionally, three bottles of reference E-liquid were prepared. Concentrations of nicotine and NRAs from each bottle were measured by HPLC. Concentrations of these alkaloids were also determined from electronic cigarette-generated aerosol and traditional cigarette smoke. Results: Nicotine concentrations in E-liquid brands 1, 2, 3, 4, 5 and in the reference E-liquid were 17.8 ± 4.1, 23.2 ± 0.7, 24.0 ± 0.9, 24.9 ± 0.2, 19.7 ± 0.3 and 20.4 ± 0.1 mg/mL, respectively. Concentrations normalized to 100% of product label were 74%, 97%, 100%, 104%, 109% and 102%, respectively. E-liquid brand 1 showed significance (*p* < 0.001) between bottles, while the reference showed the least variability. Similar results were obtained for the NRAs. Results also indicated the NRAs in aerosol of the reference E-liquid are lower than in cigarette smoke. Conclusions: The amounts of NRAs present in E-liquids and E-liquid aerosol are less compared to cigarettes, however, inconsistencies and variation in nicotine concentrations supports the need for regulatory oversight.

## 1. Introduction

The ingredients found in electronic cigarette (ECIG) refill solutions (E-liquids) are few, and for the most part, non-toxic. They include propylene glycol, glycerol, nicotine, and various flavorings [1,2,3]. While some contaminants have been detected in E-liquids and ECIG-generated aerosol, thousands of compounds, many of which are known to promote carcinogenesis, have been identified in cigarettes and cigarette smoke [4]; specifically trace metals, polycyclic aromatic hydrocarbons, volatile organic compounds and the tobacco specific N-nitrosamines (TSNA). The TSNAs are of interest since they derive from nicotine and nicotine-related alkaloids (NRAs). Nornicotine, anabasine and anatabine are minor NRAs present in tobacco and are precursors to TSNAs [5]. Since synthetic nicotine is costly to produce [6], most E-liquid solutions contain nicotine extracted from tobacco [7], bringing into question the potential for carcinogenicity. Consequently, E-liquid manufacturers are under scrutiny concerning purity and accuracy of nicotine as listed on the product label [1,8,9,10,11,12,13,14,15]. Using high performance liquid chromatography with photodiode array detection (HPLC-PDA), concentrations of nicotine and NRAs (i.e., nornicotine, cotinine, anabasine, anatabine and myosmine) from five brands of E-liquids were determined and compared to the printed concentration on the label. In addition, using a methanol extraction procedure, concentrations of all alkaloids in aerosol and smoke were determined. The results of this study confirm the work of others [1,9,16,17] who indicated that NRAs in E-liquid and ECIG-generated aerosol are in substantially lower levels than in cigarettes and conventional cigarette smoke. The advantage of the methodology used in this study is that the extraction procedure for aerosol and smoke, although less efficient in terms of percent recovery, is simple (i.e., few methodological steps), clean (i.e., reduced noise and interference in the chromatographic analysis) and fast (i.e., <2 hours/sample) and HPLC-PDA can accurately measure NRAs at extremely low levels. In practical terms, this means that the improved ability to measure extremely low levels of NRAs compensates for their low percent recovery from aerosol and smoke.

## 2. Materials and Methods

### 2.1. E-Liquids and Chemicals

Five different brands of E-liquids (indicated as E-liquid 1, 2, 3, 4 and 5) were purchased locally. Three bottles of reference E-liquid were prepared in-house consisting of propylene glycol, glycerol and (S)-(-)-nicotine, 99% (Alpha Aesar, Tewksbury, MA, USA). A detailed description of each E-liquid brand is shown in Table 1. All reagents were purchased from Fisher Scientific (Waltham, MA, USA) unless otherwise noted.

### 2.2. E-Liquid Sampling

Samples from each bottle of E-liquid were diluted 1:10 with methanol and analyzed using HPLC-PDA (details below). Each bottle was analyzed in triplicate and average values of nicotine and NRAs were determined for each bottle. The averages of each alkaloid from each bottle were subsequently determined for each E-liquid brand using the average of the three replicates from each bottle. 

### 2.3. Aerosol and Smoke Extraction

Triple 3 eGo^®^ devices purchased locally were used to aerosolize reference E-liquid. As previously described [18], these devices consisted of a 650 mAh lithium ion battery (3.7 V, unregulated), and a glass tank with a 1.6 ml capacity to house E-liquid. The resistance of the tanks’ heating coils varied between 2.2 and 2.6 Ω for an average output of 5.7 watts. Marlboro^®^ Red full strength cigarettes (84–85 mm, containing 0.671 ± 0.005 g tobacco) were combusted to created smoke. Two Cole-Parmer (Vernon Hills, IL) Master Flex L/S peristaltic pumps (one for aerosol and one for smoke) transported mainstream ECIG-generated aerosol or cigarette smoke. Master Flex L/S 24 Precision Tubing (length = 40 cm; ID = 6.4 mm) from each pump was connected to 10 cm of Tygon S3 flexible tubing (ID = 3.175 mm, OD = 4.762 mm) using a plastic connector. The Tygon tubing was connected to a Whatman 25 mm Swin-Lok^®^ plastic in-line filter holder which housed a Steritech glass fiber filter disc (25 mm diameter; 0.6 µm pore size) to trap incoming aerosol or smoke. The filter holders were loosened one half-turn to relieve in-line pressure and vent excess aerosol or smoke. Pump flow rates (400 mL/minute) simulated airflow during a five second puff. The glass filter discs were exposed to aerosol (average of three replicates per bottle of reference E-liquid; n = 3) or smoke (n = 8) for 45 cycles of a five second puff (pump on) followed by a 10 second rest period (pump off). Forty-five puffs on the ECIG device approximates three cigarettes and is equivalent to 420 µL of aerosolized E-liquid [18]. The E-liquid contained 20 mg/mL of nicotine or 2.8 mg nicotine/cigarette equivalent. A Marlboro^®^ Red cigarette contains 12.1 mg nicotine/cigarette [19] with a yield of 0.92 mg/cigarette [20]. All aerosol and smoke extractions were conducted within a P20 Purair (Airscience, Fort Myers, FL, USA) ductless fume hood equipped with HEPA filters. 

At the end of each exposure, glass fiber disks were removed from the filter holders, placed into 25 mL Erlenmeyer flasks containing 10 mL of methanol, gently shaken for 60 min, and filtered through glass fiber mesh. For percent recovery determinations, 420 µL of reference E-liquid was added to the methanol in the Erlenmeyer flasks or absorbed directly onto the disks before placing into the Erlenmeyer flasks. The flasks were shaken, filtered through glass fiber mesh and all samples were analyzed for nicotine and NRAs using HPLC-PDA (Shimadzu, Columbia, MD, USA) [9,21,22]. 

### 2.4. HPLC Analysis

**Figure 1:**
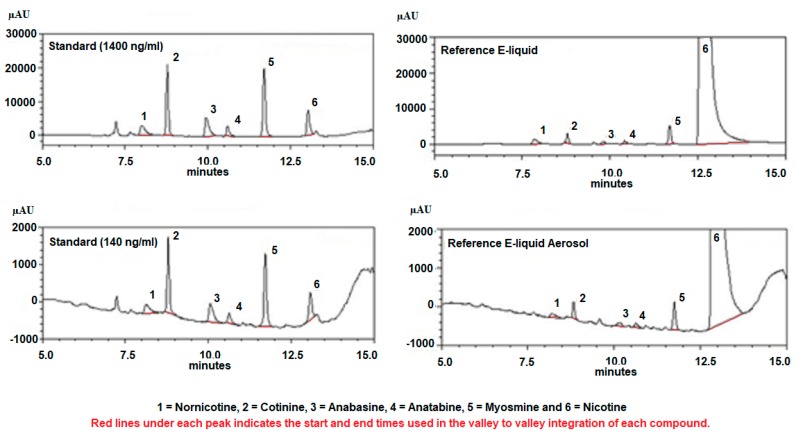
Representative chromatograms of standards and reference E-liquid and E-liquid aerosol.

A Shimadzu HPLC system (Columbia, MD, USA) was used to quantitate nicotine and NRAs, and included the following: a photodiode array detector (SPD-M20A), dual pumps (LC-20AT), a column oven (CTO-20A), an in-line membrane degasser (DGU-20A3R) and a Rheodyne 7725I manual injector with 20 µL loop (40 µL injection volume). Nicotine and NRAs were separated on a Kinetex^®^ (Phenomenex, Torrance, CA, USA) 15 cm, 5 µm reversed phase C-18 column preceded by a Security Guard (Phenomenex, Torrance, CA, USA) column. Column temperature was maintained at 35 °C. Nicotine and NRAs were detected at UV wavelengths between 230 and 300 nm and quantifications were carried out at 260 nm. The mobile phase was delivered at a rate of 1 mL/minute in gradient fashion where mobile phase A consisted of 10% acetonitrile in 20 mM ammonium formate adjusted to pH 8.5 with 50% ammonium hydroxide and mobile phase B consisted of 100% acetonitrile. Mobile phase A decreased from 100% to 80% from 0 to 10 min, decreased from 80% to 20% from 10 to 20 min, increased from 20% to 100% from 20 to 21 min and remained at 100% till the end of the run time at 30 min. Mobile phase B increased from 0% to 20% from 0 to 10 min, increased from 20% to 80% from 10 to 20 min, decreased from 80% to 0% from 20 to 21 min and remained at 0% till the end of the run time at 30 min. Standard solutions of (S)-(-)-nicotine, 99%; DL-nornicotine, 98%; (-)-cotinine, 98%; (-)-anabasine, 94%; myosmine, 98% (Alpha Aesar, Tewksbury, MA, USA); and DL-anatabine, 97% (Matrix Scientific, Columbia, SC) were prepared in 10% acetonitrile at concentrations of 1400, 700 and 140 ng/ml. Standard curves were linear (R^2^ > 0.997) and retention times for all alkaloids ranged between 8.01 and 13.08 min. Limits of detection (LOD) and limits of quantitation (LOQ) were calculated based on the calibration curve [23]. Peak identity was confirmed using absolute retention times of the closest peak (± 5%). Chromatographic integration parameters were PC-controlled using a Lab Solutions work station (Columbia, MD) and consisted of a peak width of 5 seconds at half height, and slope change from base line > 500 µV/minute. The reason for using gradient chromatography was to more quickly elute unwanted peaks that appear late in the sample chromatographic runs (at this time we do not know what compounds these peaks represent). This is the reason why chromatographic run times were allowed to continue for 30 min, even though nicotine eluted well before 15 min. Because the shift from mobile phase A to mobile phase B and back to mobile phase A caused the base line to drift, a valley to valley approach was used in the integration process. This baseline drift becomes most significant following the elution of nicotine. While the base line drift appears significant at higher gains (i.e., 0 to 2000 µAu for the 140 ng/mL standards) at lower gains (i.e., 0 to 30,000 µAu for the 1400 ng/mL standards) the drift appears negligible. Figure 1 illustrates representative standard and sample (i.e., reference E-liquid and E-liquid aerosol) chromatograms, and Table 2 outlines the standard curve statistics. 

### 2.5. Statiscal Analysis

Means ± standard errors of the mean (SEM) were calculated for nicotine and the NRAs from three replicates of each E-liquid bottle (3 bottles for each brand). One-way ANOVA, followed by Tukey’s post hoc analysis was used to detect differences between bottles of the same brand where *p* < 0.05 indicated significance.

## 3. Results

### 3.1. Nicotine and NRAs in E-Liquids

The concentrations of nicotine and NRAs in five brands of E-liquids are shown in Table 3. Nicotine in E-liquids 1, 2, 3, 4, 5 and in the reference E-liquid are 17.8 ± 4.1, 23.2 ± 0.7, 24.0 ± 0.9, 24.9 ± 0.2, 19.7 ± 0.3 and 20.4 ± 0.1 mg/mL, respectively. Concentrations normalized to 100% of product label are 74%, 97%, 100%, 104%, 109% and 102%, respectively. E-liquid 1 showed significance (*p* < 0.001) among bottles, while the reference E-liquid showed the least variability as indicated by lower coefficients of variation. Nornicotine in all bottles ranged from 5 µg/mL (E-liquid 2) to 22 µg/mL (E-liquid 5) while cotinine ranged between 1 µg/mL (E-liquid 2) to 16 µg/mL (reference E-liquid). Anabasine concentrations were undetectable in E-liquids 1, 3 and 4, while the highest levels were reported for E-liquid 5. Anatabine concentrations in E-liquid 1 displayed significance among bottles (*p* < 0.05); were undetectable in E-liquids 2 and 3; and were highest in E-liquid 5. Myosmine concentrations in E-liquid 1 displayed significance among bottles (*p* < 0.001); and ranged from 2 (E-liquids 1 and 3) to 40 ug/mL (E-liquid 4).

### 3.2. Nicotine and NRAs in Aerosol and Smoke

The concentrations of nicotine and NRAs in the aerosol generated from reference E-liquid and in smoke generated from Marlboro^®^ Reds are shown in Table 4. The amounts of all NRAs in the aerosol were lower than in the smoke. As a percent of nicotine, the NRAs ranged between 0.00 and 0.16% for the reference E-liquid and its aerosol. Using data mined from the literature [19,24,25,26,27,28,29], the NRAs, as a percent of nicotine, ranged between 0.38 and 1.45% for tobacco and its smoke. Nornicotine in the ECIG-generated aerosol was undetectable, but the percent recoveries of nicotine, cotinine, anabasine, anatabine and myosomine in the reference E-liquid aerosol were 6.66, 6.58, 6.03, 7.02 and 7.57, respectively.

## 4. Discussion

The nicotine concentrations from five brands of E-liquids range from 74 to 110% of what is printed on the product label. Furthermore, variations in nicotine concentrations exist between bottles of the same E-liquid. These results are characteristic of what others have reported [1,8,9,10,11,12,13,14,15]. This report confirms the presence of NRAs in commercially prepared E-liquids at levels similar to those previously reported, both in absolute value [14,17] and as a percent of nicotine [12,17]. The most likely antecedent of these minor alkaloids is undoubtedly the nicotine source used in the preparation of these E-liquids. The nicotine used in E-liquid preparations is typically extracted from tobacco and a small amount of NRAs are inevitably co-extracted. However, depending on the extraction method, the purity of nicotine will vary, thus yielding variable concentrations of NRAs. As suggested by Etter et al. [12], the presence of higher levels of NRAs could result from oxidative degradation of nicotine during the production of nicotine or the final E-liquid preparation. While the amounts of NRAs present in the E-liquids are low, as compared to cigarettes, the inconsistencies and excess variation in nicotine concentrations, at least in some brands, supports the current FDA regulations already in place concerning E-liquid production [30]. Given that some NRAs are precursors to the carcinogenic TSNAs [5], care must be exercised when manufacturing E-liquids.

Our HPLC system is optimized to measure low levels of tobacco alkaloids (from 54 ng/mL for norniotine to 369 ng/mL for nicotine). The aerosol extraction method used in this investigation is able to recover between 6.03 and 7.57% of all alkaloids measured, except for nornicotine which is undetectable. It is unclear why extracted nornicotine is undetectable. Possibly, the already low concentration of nornicotine in E-liquid (11.47 ± 0.48 ug/mL) does not readily elute from the glass fiber disc, thereby making it undetectable in the extract. It is well known that glass surfaces consist of silanol groups [31] and it is suspected that nornicotine binds to these silanol groups [32].The fact that only 48% of nornicotine is recovered (compared to 82–95% for the other alkaloids) when 420 µL of E-liquid is absorbed directly onto the glass fiber disk supports this conjecture. Furthermore, Yang et al. [32] report that a methanol extraction procedure for subsequent gas chromatography analysis can yield between 90 to 95% recoveries for nicotine, anabasine, anatabine and myosmine, but only 60 to 70% recovery for nornicotine. They go on to state that when less polar solvents were used in place of methanol (i.e., acetone or dichloromethane) higher than 95% recoveries were achieved for all alkaloids, including nornicotine. This indicates, at least in part, the possibility that the polarity of methanol may cause nornicotine to protonate more than the other alkaloids. Although we did not measure the content of tobacco alkaloids in cigarettes, using values mined from the literature [19,24,25,26,27,28,29], estimated percent recoveries for nicotine and NRAs ranged from 2.09% (anatabine) to 29.78% (anabasine). Despite the low percent recoveries of the minor alkaloids extracted from aerosol, the amounts that are recovered as a percent of nicotine (0.07% to 0.16%) remain remarkably close to those determined from the E-liquid outright, indicating that our extraction system can be used reliably to determine concentrations of most NRAs in aerosol. This is in agreement with Etter et al. [12] and Liu et al. [17] who reported similar levels of minor alkaloids (as a percent of nicotine) in some brands of E-liquids. In contrast, the amount of nicotine-related alkaloids extracted from cigarette smoke (as a percent of nicotine) are higher (0.38% to 1.45%) and are comparable to the results obtained by Sheng et al. [33]. Additionally, the transfer efficiencies of NRAs (i.e., the ratio of NRAs as a percent of nicotine before and after aerosolization) from E-liquid to aerosol ranges from 80 to 115%, which is comparable to the 80 to 110% obtained by Flora et al. [34].

NRAs have not typically been determined by HPLC-PDA (i.e., a UV detection at multiple wavelengths) due to the lack of sensitivity this methodology provides in determining low levels of nicotine impurities present in tobacco smoke (and even lower levels reported for E-liquids and ECIG-generated aerosol). The method of choice for determining NRA concentration is usually HPLC or gas chromatography (GC) linked to mass spectrometry (MS) detection [1,12,16,17,19,28,29,34,35,36]. To our knowledge, only one other study [9] has attempted to measure NRAs in both smoke and in ECIG aerosol using PDA as a means of detection. In their study, the order of elution for nicotine and NRAs (retention times ranging from six to 18 min) were similar to what is reported in this investigation, but the limits of detection (LODs) and quantitation (LOQs) for the NRAs are higher than what is reported in the current study, and consequently, their NRA concentrations in aerosol were mostly undetectable. On the other hand, they were able to measure NRAs in cigarette smoke. Table 5 compares the methodologies used and concentrations achieved for NRAs in E-liquid and in the aerosol of the current study with that of other studies. From this table, the NRA concentrations in both the E-liquid and aerosol compare favorably with what has previously been reported (i.e., all concentrations of NRAs are in the low µg range), even though the methodologies used to determine the NRAs differ.

Although the NRA concentrations reported in this investigation agree favorably with those previously reported, there are limitation that need to be addressed. The first limitation is that the NRAs in the extracted aerosol is recovered at a paltry 6–7%. Better methods for NRA extraction from aerosol and subsequent measurement have be used by others [1,9,16,17,34] of which several follow CORESTA guidelines [37]. The outcome is that a more accurate and precise determination of NRAs in aerosol is achieved. However, these methodologies often use special equipment such as impingers for sparging, expensive aerosol traps, smoking machines (for more precise puff topography), and most likely require mass spectrometry as a means of measurement. The aforementioned equipment can be costly, both in terms of money and time. The extraction and measurement methodologies used in the current investigation, albeit not as accurate or precise, were able to achieve comparable NRA concentrations as those previously reported both in E-liquid and ECIG aerosol (see Table 5), minus the expense. Another limitation is that a direct measurement of NRAs in ECIG aerosol was used in this study rather than the indirect method of mass change tracking (MCT). The MCT approach [38,39,40] measures the amount of analyte (in this case the NRAs) in a known quantity of E-liquid and tracks how much is lost through the vaporization process. Thus, the indirect MCT approach assumes all the analyte that is lost must therefore be vaporized. This is a means of accounting for any analyte that is lost (e.g., through condensation in the pump tubing or loss through venting of the inline chamber) when using the direct approach to analyte measurement. Condensation and venting of aerosol are the most likely reasons for the low NRA percent recoveries reported. On the other hand, we could have used the MCT approach since we already knew (from previous work published from this laboratory) that for each puff, 9.3 µL of e-liquid is vaporized, such that after 45 puffs a total of 420 µL is vaporized [18]. The units could also be easily converted to µg/g of E-liquid since it is known that 420 µL of E-liquid is equal to 448.4 mg [18], but opted to go with µg/mL instead. In retrospect, it would have been possible to determine the total amount of NRAs in a full tank of E-liquid and subsequently determine the amount of NRAs in the tank as the volume is decreased by puffing. With that said, our HPLC-PDA methodology was optimized to measure concentrations of NRT in the E-liquid. To our knowledge, this is the first time that UV detection has been used to quantitate NRAs in E-liquid. Furthermore, since we expected a high transfer efficiency (between 80 to 115%) of NRAs from the E-liquid to the aerosol, [34] the low % recovery becomes less important than the fact that we could measure these NRAs from 420 µL of E-liquid and assume nearly 100% transfer. Finally, this study did not employ CORESTA guidelines [37] when implementing puff topography. While this strays from current efforts to establish uniformity in studies investigating ECIG-generated aerosol across labs, we decided to use puff parameters already established in this laboratory to be consistent with other outputs from this laboratory. The puffing regimen used in our studies are similar to one used by Goniewicz et al. [13] in 2013, well before the CORESTA guidelines were published in 2015. Regardless of these limitations, our results are comparable to those previously published and provide a simple, clean and fast means for NRA quantitation in E-liquids and ECIG-generated aerosol.

## 5. Conclusions

In conclusion, our extraction procedure, coupled with HPLC-PDA, can effectively determine low-level NRAs in aerosol and smoke. The amounts of NRAs present in E-liquids, along with their emissions, are much lower compared to Marlboro^®^ Red cigarettes, confirming previously published reports. However, inconsistencies and excess variation in nicotine concentrations, at least in some brands of E-liquids, support the need for continued regulatory oversight. 

## Figures and Tables

**Table 1:** Description of E-liquids used in study.

E-Liquid	Date	Batch	Expiration Date	Ratio of PG/VG	Stated	Additional
Brand	Purchased or Acquired	Identification			Level of Nicotine	Ingredients
E-liquid 1						
Bottle 1	Jul 18, 2016	Not Available	Not Available	80/20	24 mg/mL	Tobacco Flavoring
Bottle 2	Jul 25, 2016					
Bottle 3	Aug 1, 2016					
E-liquid 2						
Bottle 1	Jul 18, 2016	16TB30E5801	Apr 2018	70/30	24 mg/mL	Tobacco Flavoring
Bottle 2	Jul 25, 2016	16TB30E9401	Jul 2018			
Bottle 3	Aug 1, 2016	16TB30E9401	Jul 2018			
E-liquid 3						
Bottle 1	Jul 18, 2016	6036CBY24	Not Available	50/50	24 mg/mL	Tobacco Flavoring
Bottle 2	Jul 25, 2016	6036CBY24				
Bottle 3	Aug 1, 2016	6036CBY24				
E-liquid 4						
Bottle 1 Bottle 2 Bottle 3	Jul 18, 2016 Jul 25, 2016 Aug 1, 2016	Not Available	Not Available	60/30	24 mg/mL	Tobacco flavoring
E-liquid 5						
Bottle 1	Jul 18, 2016	VSEJ	Nov 2017	70/30	18 mg/mL	Tobacco Flavoring
Bottle 2	Jul 25, 2016	VSEJ	Nov 2017			
Bottle 3	Aug 1, 2016	VSEJ	Nov 2017			
Reference						
Bottle 1	Aug 11, 2016	Batch 1	Not Available	50/50	20 mg/mL	No Flavorings
Bottle 2	Aug 11, 2016	Batch 2				
Bottle 3	Aug 11, 2016	Batch 3				

PG = propylene glycol; VG = vegetable glycerin (i.e., glycerol).

**Table 2:** Standard curve statistics.

Compound	Injected Concentration (ng/mL)	Retention Time (min)	Measured Concentration (ng/mL)	Area Under Peak	Straight Line Equation	LOD and LOQ (ng/mL) *	Lowest X Value Used (ng/mL) **	R^2^	% RSD
Nicotine	1400.00 ng/mL	13	1409	45,034	Y = 32X − 560	122 and 369	150,203	1	6.7
	700.00 ng/mL	13.1	658	20,573					
	140.00 ng/mL	13.1	164	4695					
Nornicotine	1400.00 ng/mL	8	1403	29,386	Y = 21X − 332	18 and 54	410	1	4.2
	700.00 ng/mL	8.1	694	14,368					
	140.00 ng/mL	8.1	143	2706					
Cotinine	1400.00 ng/mL	8.8	1393	97,916	Y = 70X + 478	46 and 139	227	1	2.4
	700.00 ng/mL	8.8	716	50,563					
	140.00 ng/mL	8.8	131	9651					
Anabasine	1400.00 ng/mL	10	1385	48,629	Y = 35X + 753	94 and 286	322	1	3.7
	700.00 ng/mL	10	733	26,100					
	140.00 ng/mL	10.1	121	4951					
Anatabine	1400.00 ng/mL	10.6	1389	18,417	Y = 13X + 193	69 and 208	416	1	2.7
	700.00 ng/mL	10.6	724	9685					
	140.00 ng/mL	10.6	127	1856					
.Myosmine	1400.00 ng/mL	11.7	1392	109,515	Y = 78X + 971	50 and 153	674	1	1.96
	700.00 ng/mL	11.7	718	56,923					
	140.00 ng/mL	11.7	1308	11,123					

* Limit of detection (LOD) and limit of quantitation (LOQ) were determined based on the calibration curve. ** Lowest X value used from all sample chromatograms. RSD = relative standard deviation (i.e., Coefficient of variation).

**Table 3:** Comparison of nicotine and nicotine-related alkaloids from various brands of E-liquid.

Compound	E-liquid 1	E-liquid 2	E-liquid 3	E-liquid 4	E-liquid 5	Reference
Stated level of nicotine (mg/mL) on label	24	24	24	24	18	20
Percent of stated nicotine on label	74%	97%	100%	104%	110%	102%
Nicotine (mg/mL) *						
Bottle 1 (n = 3)	22 ± 2 ^b^	25 ± 1	25 ± 1	25 ± 1	20 ± 1	20 ± 0
Bottle 2 (n = 3)	10 ± 0 ^a,c^	22 ± 0	25 ± 0	25 ± 0	19 ± 0	20 ± 0
Bottle 3 (n = 3)	22 ± 1 ^b^	22 ± 2	22 ± 2	25 ± 1	19 ± 2	21 ± 0
**Mean ± SEM (n = 3) #**	**18 ± 4**	**23 ± 1**	**24 ± 1**	**25 ± 0**	**20 ± 0**	**20 ± 0**
**% RSD (n = 9) @**	**36**	**9**	**10**	**4**	**11**	**1**
Nornicotine (µg/mL) *						
Bottle 1 (n = 3)	14 ± 3	5 ± 0	18 ± 0	13 ± 1	21 ± 0	18 ± 0
Bottle 2 (n = 3)	11 ± 0	5 ± 0	16 ± 0	15 ± 0	22 ± 0	17 ± 0
Bottle 3 (n = 3)	15 ± 2	6 ±1	20 ±1	12 ± 1	19 ± 2	17 ± 1
**Mean ± SEM (n = 3) #**	**13 ± 1**	**5 ± 0**	**18 ± 0**	**13 ± 0**	**21 ± 0**	**17 ± 0**
**% RSD (n = 9) @**	**29**	**16**	**11**	**11**	**10**	**4**
Cotinine (µg/mL) *	3 ± 1			undetectable	undetectable	
**Bottle 1 (n = 3)**	undetectable	1 ± 0	4 ± 1	2 ± 0	2 ± 0	15 ± 0
**Bottle 2 (n = 3)**	3 ± 1	undetectable	2 ± 0	1 ± 1	1 ± 1	15 ± 0
**Bottle 3 (n = 3)**		undetectable	2 ± 1			16 ± 0
**Mean ± SEM (n = 3) #**			**3 ± 1**			**15 ± 0**
**% RSD (n = 9) @**			**47**			**5**
Anabasine (µg/mL) *						
Bottle 1 (n = 3)	undetectable	3 ± 0	undetectable	undetectable	69 ± 5	7 ± 1
Bottle 2 (n = 3)	undetectable	2 ± 0	undetectable	undetectable	70 ± 1	8 ± 0
Bottle 3 (n = 3)	undetectable	2 ± 0	2 ± 0	2 ± 0	58 ± 5	8 ± 0
**Mean ± SEM (n = 3) #**		**2 ± 0**			**66 ± 4**	**8 ± 0**
**% RSD (n = 9) @**		**20**			**13**	**9**
Anatabine (µg/mL) *						
Bottle 1 (n = 3)	286 ± 39 ^e,f^	1 ± 0	undetectable	288 ± 13	817 ± 37	15 ± 0
Bottle 2 (n = 3)	138 ± 3 ^d^	undetectable	undetectable	286 ± 3	841 ± 21	15 ± 0
Bottle 3 (n = 3)	176 ± 8 ^d^	undetectable	undetectable	268 ± 15	847 ± 118	14 ± 0
**Mean ± SEM (n = 3) #**	**203 ± 48**			**279 ± 6**	**835 ± 9**	**15 ± 0**
**% RSD (n = 9) @**	**37**			**7**	**13**	**3**
Myosmine (µg/mL) *						
**Bottle 1 (n = 3)**	30 ± 3 ^g,h^	4 ± 0	3 ± 0	40 ± 2	14 ± 0	14 ± 0
**Bottle 2 (n = 3)**	2 ± 0 ^i^	4 ± 0	3 ± 0	38 ± 0	14 ± 0	14 ± 0
**Bottle 3 (n = 3)**	7 ± 0 ^i^	3 ± 1	2 ± 1	33 ± 3	14 ± 2	15 ± 1
**Mean ± SEM (n = 3) #**	**13 ± 9**	**4 ± 0**	**3 ± 0**	**37 ± 2**	**14 ± 0**	**14 ± 0**
**% RSD (n = 9) @**	**99**	**15**	**36**	**12**	**13**	**3**

*= Mean ± SEM for each bottle. # = Mean ± SEM for three bottles. @ = Coeffiecient of variation for all nine bottles a = Significant (*p* < 0.001) from bottle 1; b = Significant (*p* < 0.001) from bottle 2; c = Significant (*p* < 0.001) from bottle 3. d = Significant (*p* < 0.05) from bottle 1; e = Significant (*p* < 0.05) from bottle 2; f = Significant (*p* < 0.05) from bottle 3. g = Significant (*p* < 0.001) from bottle 1; h = Significant (*p* < 0.001) from bottle 2; i = Significant (*p* < 0.001) from bottle 3. RSD = relative standard deviation (i.e., coefficient of variation). Bold section represents the take home values of Mean± SEM and the % RSD for each E-liquid.

**Table 4:** Nicotine and nicotine-related alkaloids generated from E-liquid aerosol and tobacco smoke.

	Reference E-liquid				Marlboro^®^ Red Cigarette		
Compound	µg/mL of 420 µL of E-liquid (3 cigarette equivalent) (n = 3) *	µg/mL of 420 µL of E-liquid (3 cigarette equivalent) on disk (n = 3) @	µg/mL of 45 puffs of E-liquid (3 cigarette equivalent) on disk (n = 3) #	% Recovery ^	µg per 3 cigarette equivalent from the primary literature !	µg in 45 puffs (3 cigarette equivalent) on disk (n = 8) #	Estimated % Recovery ^
Nicotine	21227 ± 37	20144 ± 1397	1414 ± 66	6.7	39480 [24]	4719 ± 637	13.0 to 14.7%
					36435 [19]		
					32700 [25]		
					36210 [26]		
					35160 [27]		
					32025 [28]		
Nornicotine	11.5 ± 0.5 (0.11%)	5.6 ± 0.9 (0.06%)	Undetectable (0.00%)	0.0	1509 [19]	68.3 ± 9.1 (1.45%)	4.5 to 8.9%
					768 [28]		
					1526 [29]		
Cotinine	15.5 ± 0.2 (0.15%)	14.2 ± 0.7 (0.14%)	1.0 ± 0.1 (0.12%)	6.6	not available	55.9 ± 8.2 (1.18%)	not determinable
Anabasine	7.8 ± 0.2 (0.07%)	6.4 ± 0.9 (0.06%)	0.5 ± 0.0 (0.07%)	6.0	143 [19]	17.9 ± 2.1 (0.38%)	6.0 to 29.8%
					60 [28]		
					296 [29]		
Anatabine	16.7 ± 0.6 (0.16%)	14.3 ± 0.9 (0.14%)	1.2 ± 0.2 (0.16%)	7.0	1107 [19]	46.2 ± 6.0 (0.98%)	2.1 to 8.5%
					546 [28]		
					2214 [29]		
Myosmine	14.1 ± 0 (0.13%)	13.2 ± 1.0 (0.13%)	1.1 ± 0.1 (0.15%)	7.6	28 [29]	40.8 ± 5.4 (0.87%)	>100%

Values given as Mean ± SEM determined from triplicates of three bottles of E-liquid or from Marlboro^®^ Red cigarette. Values of in parenthesis represent alkaloid as a percent of nicotine. * = 420 µL of E-liquid is equivalent to amount vaporized in 45 puffs (i.e., 3 cigarette equivalent). @ = 420 µL is placed on a glass fiber disk and extracted in10 ml of methanol. # = 45 puffs aerosol or smoke extracted from glass fiber disks in 10 ml of methanol. ^ = percent of aerosol or smoke (based on total content) recovered from glass fiber disks. ! = Values found in the literature and adjusted to reflect the content in three cigarettes using 0.671 ± 0.005 g as the weight of tobacco in a Marlboro^®^ Red cigarette.

**Table 5:** Comparison of methodologies and concentrations for nicotine-related alkaloids in (**A**) E-liquids and (**B**) electronic cigarette (ECIG)-generated aerosol of the current study compared with other studies.

**A. NRAs in E-liquids**											
**Study**	**E-Liquid Brand**				**Extraction Process**	**Measured by**	**Nornicotine**	**Contine**	**Anabasine**	**Anatabine**	**Myosmine**
Current Study	Various refill solutions				420 µL of E-liquid diluted in 10 mL of methanol	HPLC-PDA	5-21	BDL-15	µg/mL BDL-66	15-835	3-37
Trehy et al. [9]	Various cartridges				Cartridge pad diluted in 50 mL of Methanol	HPLC-PDA	NA	BDL	µg/cartridge BDL	BDL-0.8	<LOQ-0.1
Etter et al. [12]	Various refill solutions				Dilution with 1 M ammonia	GC-MS or GC-flame ionization	BDL-15	BDL-39	µg/mLBDL-83	BDL-374	BDL-77
Liu et al. [17]	Laboratory Reference E-liquid				1 to 10 dilution in 100 mM ammonium acetate	LC-MS/MS	5	31	µg/g BDL	23	6
Flora et al. [34]	Various Cartridges and refill Solutions				Dilution with 70% Methanol	LC-MS	<48	<48	µg/mL #<48	<144	<48
B. NRAs in ECIG-generated aerosol											
**Study**	**Puff Volume (mL)**	**Number of Puffs**	**Puff /Interval (s)**	**BatteryPower (W)**	**Extraction Process**	**Measured by**	**Nornicotine**	**Contine**	**Anabasine**	**Anatabine**	**Myosmine**
Current Study	33.6	45	5/10	5.7	Methanol extraction from a glass fiber disk	HPLC-PDA	BDL	16	µg/mL @ 8	17	14
Trehy et al. [9]	100	30	?/60	?	10% acetonitrile in water using an impinge	HPLC-PDA	NA	BDL	µg/cartridge BDL	<LOQ	BDL
Margham et al. [16]	55	200	3/30	4.5	ammonium acetate extraction from glass fiber disk *	HPLC-MS/MS	BDL	1	µg/collection <LOQ	<LOQ	3
Liu et al. [17]	55	50	3/30	5	ammonium acetate extraction from glass fiber disk *	HPLC-MS/MS	0.9	NA	µg/collection 2.1	6.0	<LOQ
Flora et al. [34]	55	100	4/30	?	extraction from glass fiber disk	LC-MS	<45-53	<49	µg/mL ! <38-44	128-138	<47

NA = not available; BDL = below detection limit or <LOD; ? = information not specified: * = used CORESTA methods [37]; # = Concentrations calculated from % of nicotine and assumed nicotine in the cartridges and refill solutions is 24 mg/ml; @ = Concentrations given are based on 100% recovery of the reference E-liquid in the current study; ! = concentrations given are based off transfer efficiencies from the concentrations already specified in part A of this table.
